# ChatSpatial: Schema-Enforced Agentic Orchestration for Reproducible and Cross-Platform Spatial Transcriptomics

**DOI:** 10.64898/2026.02.26.708361

**Published:** 2026-03-09

**Authors:** Chen Yang, Xianyang Zhang, Jun Chen

**Affiliations:** 1 Department of Statistics, Texas A&M University, College Station, Texas, 77843, USA; 2 Division of Computational Biology, Department of Quantitative Health Sciences, Mayo Clinic, Rochester, Minnesota, 55905, USA

**Keywords:** spatial transcriptomics, artificial intelligence, bioinformatics integration, natural language processing, computational biology, software platform, cancer research, MCP, agentic orchestration

## Abstract

Spatial transcriptomics has transformed our ability to study tissue architecture at molecular resolution, yet analyzing these data demands navigating dozens of computational methods across incompatible Python and R ecosystems—forcing researchers to devote more effort to making tools function than to pursuing biological questions. We present ChatSpatial, a platform in which the LLM selects from pre-validated tool schemas rather than generating free-form code, with domain expertise embedded in schema descriptions for context-aware parameter inference. Built on the Model Context Protocol (MCP), ChatSpatial unifies 60+ methods across 15 analytical categories into a single conversational workflow spanning Python and R ecosystems. Replication of two published studies—recovering subclonal heterogeneity in ovarian cancer and tumor microenvironment organization in oral squamous cell carcinoma—and validation across seven LLM platforms demonstrate that schema-enforced orchestration yields near-deterministic reproducibility at the workflow level for multi-step spatial analyses. Beyond replication, exploratory cross-method analyses illustrate practical triangulation across independent analytical frameworks.

## 1 Introduction

Spatial transcriptomics is transforming biology by revealing how cellular identity and function are shaped by tissue architecture [1, 2]. Yet while spatial technologies generate data at an unprecedented scale, the technical expertise required to integrate fragmented computational ecosystems has become the rate-limiting step for discovery. The widespread adoption of platforms like 10x Genomics Visium has shifted the primary bottleneck from data acquisition to computational analysis, leading to a rapid proliferation of analytical tools [3]. This growth, however, has produced a fragmented ecosystem divided between Python and R—built on incompatible data objects (Ann-Data [4] and Seurat [5])—that translates into practical, time-consuming hurdles for biomedical researchers.

Consider a cancer biologist who wishes to identify spatial domains using SpaGCN (Python) and then analyze cell-cell communication using CellChat (R). This straight-forward scientific goal requires managing conflicting software environments, writing custom data conversion scripts, and manually integrating outputs—a significant barrier for researchers without specialized programming expertise. A recent Comment in *Nature Biotechnology* identified similar issues—fragmented documentation, lack of reproducibility, and high technical barriers—as primary obstacles to adopting computational tools in single-cell biology [6].

Recent reviews have identified computational analysis as “a major challenge facing spatial transcriptomics,” noting that tools are “often difficult to evaluate and apply” due to algorithmic complexity and the requirement for coding expertise [7, 8]. To address these barriers, several strategies have emerged: “bridge” tools for cross-ecosystem data conversion (e.g., zellkonverter), and more recently, LLM-powered systems ranging from interactive co-pilots [9, 10] to multimodal semantic exploration tools [11] to fully autonomous agents [12, 13]. However, autonomous agents pose reproducibility challenges due to non-deterministic code generation, while co-pilots typically assist with specific tasks within a single ecosystem. This leaves an important gap for researchers who need a system that combines the flexibility of natural language with the control and reproducibility required for complex, cross-platform workflows.

Here, we present ChatSpatial, a platform built on **schema-enforced orchestration**: the LLM selects from pre-validated tool schemas rather than generating free-form code, improving reproducibility at the workflow level in a domain where traditional LLM code generation exhibits substantial hallucination rates [14–16]. Our contribution is threefold: (1) schema-enforced orchestration as an architectural paradigm—built on the Model Context Protocol (MCP) [17]—in which the LLM acts as a reliable orchestrator rather than an autonomous agent or code generator, with domain expertise embedded directly in schema descriptions for context-aware parameter inference (Methods §4.1); (2) a comprehensive cross-ecosystem implementation unifying 60+ methods across Python and R through a single conversational workflow, eliminating the technical overhead of environment management and data conversion; and (3) systematic validation through replication of two published spatial transcriptomics studies and cross-model reproducibility experiments across seven LLM platforms. This design allows researchers with basic command-line familiarity to execute end-to-end spatial analyses through natural language, enabling iterative exploration while keeping the scientist in full control.

Unlike fully autonomous systems that prioritize end-to-end automation [12, 13], ChatSpatial is designed for human-steered discovery—prioritizing analytical fidelity and reproducibility while the researcher retains strategic control (Table 1). In this work, we demonstrate how this approach enables complex cross-platform analytical strategies through a conversational workflow.

## 2 Results

### 2.1 Overview of the ChatSpatial Platform

**Fig. 1:**
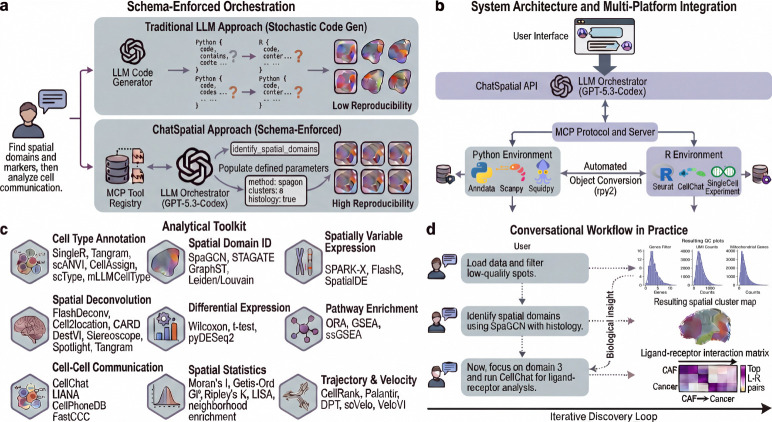
ChatSpatial eliminates the implementation tax in spatial transcriptomics analysis through MCP-based tool orchestration. (**a**) Schema-enforced orchestration versus traditional code generation. Top: conventional LLM approaches generate free-form code with stochastic outputs, leading to low reproducibility due to hallucinated functions and incompatible API calls. Bottom: ChatSpatial constrains LLM outputs to pre-validated tool schemas via the Model Context Protocol (MCP), ensuring that user intent maps to defined parameter spaces with high reproducibility. (**b**) System architecture and multi-platform integration. User queries pass through the ChatSpatial API to an LLM orchestrator, which invokes tools via the MCP Protocol and Server layer. Automated object conversion (rpy2) bridges the Python environment (AnnData, Scanpy, Squidpy) and R environment (Seurat, CellChat, SingleCellExperiment), enabling seamless cross-ecosystem workflows. (**c**) Analytical toolkit spanning 9 major categories and 60+ methods: cell type annotation (SingleR, Tangram, scANVI, CellAssign, scType, MLLM-CellType), spatial domain identification (SpaGCN, STAGATE, Leiden/Louvain), spatially variable expression (SPARK-X, FlashS, SpatialDE), spatial deconvolution (FlashDeconv, Cell2location, CARD, DestVI, Stereoscope, Spotlight, Tangram), differential expression (Wilcoxon, t-test, pyDESeq2), pathway enrichment (ORA, GSEA, ssGSEA), cell-cell communication (CellChat, LIANA, CellPhoneDB, FastCCC), spatial statistics (Moran’s *I*, Getis-Ord, Ripley’s *K*, LISA, neighborhood enrichment), and trajectory & velocity (CellRank, Palantir, DPT, scVelo, VeloVI). (**d**) Conversational workflow in practice. An iterative discovery loop demonstrates three sequential prompts—data loading with QC, spatial domain identification, and ligand-receptor analysis—producing publication-quality results and biological insights (e.g., CAF→Cancer interactions) without manual scripting.

ChatSpatial transforms spatial transcriptomics analysis from a programming challenge into a scientific conversation (Figure 1a). All case study analyses and the 28 validation scenarios were conducted using a single LLM (Claude Sonnet 4.5 [18], model ID claude-sonnet-4–5-20250929) to ensure consistency; the cross-model experiments in §2.3 additionally tested Gemini 2.5 Flash and GPT-5 Mini to assess model-agnostic reproducibility. The platform coordinates 60+ methods across 15 major analytical categories, including data preprocessing, integration, cell type annotation, spatial domain identification, cell communication, and deconvolution (Figure 1b–c). By accepting natural language queries, ChatSpatial enables an iterative conversational workflow that executes complex cross-ecosystem pipelines without requiring users to implement the underlying integration code (Figure 1d). This interaction model shifts the user’s focus from technical implementation to biological interpretation.

These analytical categories reflect established frameworks for spatial transcriptomics computational analysis [3, 19]. Method selection within each category prioritized benchmark-validated performance [20–24], broad community adoption [25–28], and computational efficiency for large datasets [29, 30]. Multiple complementary methods are deliberately included within each category to accommodate distinct data modalities (e.g., with versus without histology images) and algorithmic approaches, enabling users to either receive context-aware recommendations from the platform or perform comparative analyses across methods.

The platform’s conversational interface maintains stateful context across analytical steps. A user can initiate a multi-step workflow with: *“Identify spatial domains in this breast cancer Visium sample, annotate the cell types using an scRNA-seq reference, and find genes marking the tumor-stroma boundary,”* then follow up with *“Now, zoom into domain 3 and perform cell communication analysis”* without re-specifying the dataset. This paradigm extends to method selection: when a user requests *“identify spatial domains”*, the platform recommends the most suitable method based on data characteristics (e.g., SpaGCN for Visium with histology, STAGATE without), while the researcher retains full control to direct comparative analyses across methods. A comprehensive set of example interactions is provided in Supplementary Table S2.

### 2.2 Case Studies: Replication and Exploratory Analysis

To validate ChatSpatial’s ability to replicate expert-level analytical workflows, and to illustrate how schema-enforced orchestration enables hypothesis-generating exploration, we applied it to two published studies representing distinct biological challenges: high-resolution tumor microenvironment analysis and multi-sample cohort characterization.

#### 2.2.1 Oral Cancer: Tumor Architecture and Cell-Cell Communication

We replicated the analytical workflow from a recent study by Arora et al. [32]. This study characterized the distinct transcriptional architectures of the Tumor Core (TC) and Leading Edge (LE) in oral squamous cell carcinoma (OSCC). The original authors employed a rigorous multi-step pipeline involving cohort integration, unsupervised spatial domain identification, reference-based deconvolution, and ligand-receptor analysis. We sought to replicate this workflow using ChatSpatial’s conversational interface.

##### Step 1: Unsupervised Spatial Domain Discovery and Cohort Integration

The workflow began with identifying the primary spatial domains. To accurately replicate the study’s cohort-level analysis, we directed the platform to process all 12 patient samples simultaneously: *“Load all 12 OSCC samples, filter low-quality spots (<200 genes), and identify spatial domains using Leiden clustering with spatially-aware neighbor graphs.”* This single instruction coordinated the complex ingestion of the entire cohort and performed unsupervised spatial domain identification on each sample. We note that the original study employed Louvain clustering with Seurat’s CCA-based integration on malignant spots only, whereas ChatSpatial used Leiden clustering on combined transcriptomic and spatial neighbor graphs across all spots—a methodological difference that did not alter the key biological conclusions, as demonstrated by the concordance analysis below.

Following the unsupervised clustering performed by the platform, we examined the top marker genes retrieved by the agent. This enabled the precise biological annotation of three distinct states described in the original study: a Tumor Core (TC) enriched for keratinization and epithelial differentiation markers (e.g., *SPRR1B*, *CLDN4*, *SPRR2E*), a Transitory state marked by *KRT6C*, and a Leading Edge (LE) enriched for partial-EMT and invasion markers (e.g., *FN1*, *LAMC2*, *ITGA5*). The molecular signatures generated by the pipeline precisely matched the definitions established by Arora et al.

##### Step 2: Deconvolution Reveals Conserved Microenvironment Composition

Next, to reproduce the cellular composition analysis, we performed deconvolution using the same reference dataset used in the original study. We prompted: *“Deconvolve cell types in the identified TC and LE domains using FlashDeconv, utilizing the HNSCC single-cell reference from Puram et al.”* [31]. This command triggered the retrieval and processing of the specific scRNA-seq reference and executed FlashDeconv across the spatial data.

**Fig. 2:**
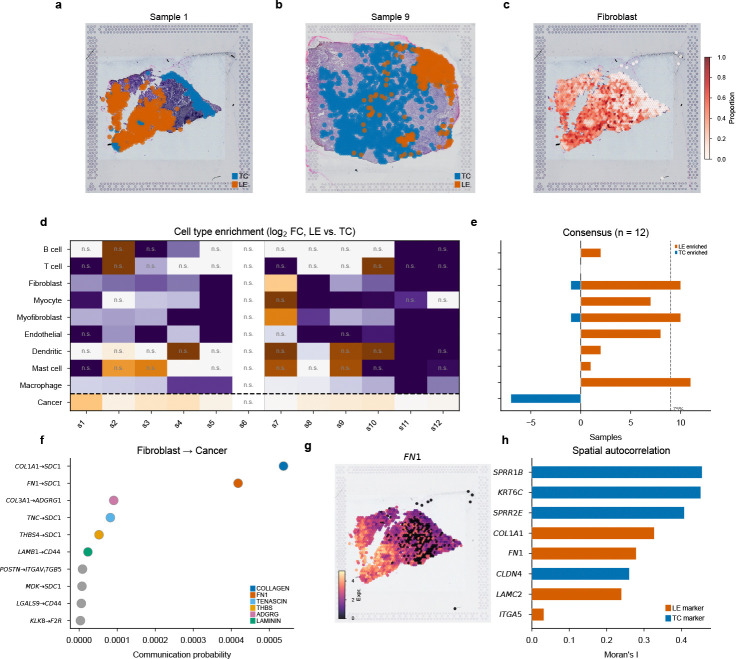
ChatSpatial replicates published OSCC tumor architecture analysis through conversational orchestration. (**a,b**) H&E tissue sections of two representative OSCC samples (Sample 1 and Sample 9 from GSE208253) with spatial domain annotations: Tumor Core (TC, blue) and Leading Edge (LE, orange). Spatial domains were identified by Leiden clustering on spatially-aware neighbor graphs and classified using marker gene scoring. (**c**) Spatial deconvolution map (Sample 1) showing fibroblast/CAF proportion at each Visium spot (FlashDeconv with HNSCC scRNA-seq reference [31]), revealing fibroblast concentration at the leading edge. (**d**) Per-sample cell type enrichment between TC and LE across all 12 samples. Color indicates log_2_ fold change (LE/TC); cells marked “n.s.” are non-significant (Mann–Whitney *U* test, BH-FDR *q* ≥ 0.05). (**e**) Consensus enrichment summary. Dashed line: 75% threshold (≥9/12 samples). Macrophages (11/12, 92%) and fibroblasts (10/12, 83%) show robust LE enrichment; cancer cells are TC-enriched (7/12, 58%). (**f**) CellChat ligand-receptor analysis identifying top Fibroblast→Cancer interactions colored by signaling pathway. Directional ECM-syndecan signaling (COL1A1→SDC1, FN1→SDC1) recapitulates the original study’s mechanistic conclusion [32]. (**g**) Spatial expression of *FN1* (Sample 1), confirming localization of the CellChat-identified ligand to the LE domain. (**h**) Moran’s *I* spatial autocorrelation for key TC and LE marker genes (Sample 1, 3,000 highly variable genes tested). TC markers *SPRR1B* (rank 6, *I* = 0.54) and LE markers *COL1A1* (rank 20, *I* = 0.50) and *FN1* (rank 23, *I* = 0.49) all rank in the top 1% of spatially variable genes.

The agent-generated results replicated the original consensus findings: the Leading Edge exhibited a distinct immune-stromal landscape compared to the Tumor Core. Specifically, ChatSpatial’s analysis revealed robust enrichment of macrophages (11/12 samples, 92%) and fibroblasts/CAFs (10/12, 83%) in the LE region, with endothelial cells (8/12, 67%) and myocytes (7/12, 58%) showing consistent LE-directional enrichment below the 75% consensus threshold (Figure 2a–e). Cancer cells were TC-enriched in 7/12 samples (58%). The core “fibrovascular niche” at the invasion front—characterized by the co-enrichment of fibroblasts and macrophages in the LE—was accurately recapitulated, matching the central finding of Arora et al. We note two methodological differences that affect quantitative comparisons. First, the use of FlashDeconv (a constrained regression approach) rather than CARD (a conditional autoregressive model) can alter estimated proportions for rarer cell types. Second, our enrichment analysis compares within-spot deconvolved proportions between TC and LE regions, whereas Arora et al. assessed cell type abundance in spots *neighboring* cancer spots—a spatial neighborhood approach that is more sensitive to low-abundance populations such as T cells. These differences explain the reduced enrichment signal for dendritic and T cells in our analysis. Nevertheless, the dominant stromal and immune signatures—fibroblasts and macrophages in the LE, cancer cells in the TC—were concordant across methods, demonstrating the platform’s ability to recover the central biological conclusions despite methodological variation.

##### Step 3: Deciphering the Interaction Mechanism via Cross-Ecosystem Orchestration

Finally, we investigated the cell-cell communication networks driving this invasion. Following the original study’s focus on tumor-stroma interactions, we utilized ChatSpatial’s ability to invoke native R tools. We issued the command: *“Focus on the Leading Edge (LE) domain and perform cell communication analysis between CAFs and cancer cells using CellChat. Specifically, look for interactions involving ECM ligands.”*

Through seamless cross-ecosystem invocation of the R-based CellChat package, the platform identified 142 significant Fibroblast→Cancer interactions spanning six signaling pathways (COLLAGEN, FN1, TENASCIN, THBS, LAMININ, and ADGRG). The top-ranked interactions—COL1A1→SDC1, COL1A2→SDC1, and FN1→SDC1—recapitulate the original study’s mechanistic conclusion that directional ECM-syndecan signaling mediates tumor progression at the invasive front (Figure 2f). Spatial expression mapping of *FN1* confirmed its localization to the LE domain (Figure 2g).

##### Beyond Replication: Cross-Method Spatial Autocorrelation

To demonstrate how schema-enforced orchestration connects findings across independent analytical frameworks, we performed a follow-up that Arora et al. did not report: spatial autocorrelation analysis (Moran’s *I*) to test whether the CellChat-identified ligands are also among the most spatially variable genes. With a single prompt—*“Run spatial autocorrelation on this sample and check whether FN1 and COL1A1 are spatially variable”*—the platform computed Moran’s *I* across 3,000 highly variable genes in Sample 1 (squidpy; permutation-based *p*-values with Benjamini–Hochberg FDR correction). Both LE markers ranked in the top 1%: *COL1A1* (rank 20/3,000, *I* = 0.50, FDR < 0.05) and *FN1* (rank 23, *I* = 0.49). The TC markers showed similarly strong spatial patterning: *SPRR1B* ranked 6th overall (*I* = 0.54), with *SPRR2E* (rank 28, *I* = 0.48) and *KRT6C* (rank 32, *I* = 0.47) in the top 1.1%. This illustrates multi-tool chaining in practice: connecting cell communication signatures to spatial gene expression rankings across two independent analytical frameworks through a single follow-up prompt (Figure 2g–h).

#### 2.2.2 Ovarian Cancer: Multi-Sample Subclone and Microenvironment Analysis

**Fig. 3:**
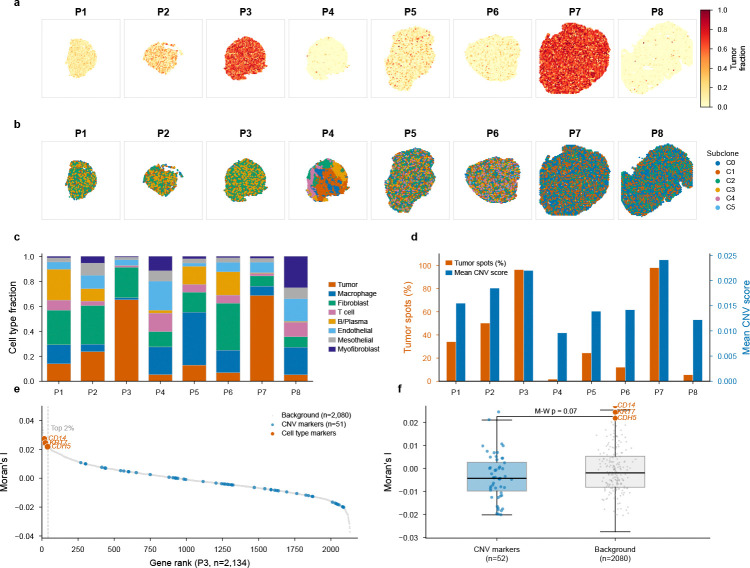
ChatSpatial replicates published HGSOC subclone analysis through multi-sample conversational workflow. Replication of Denisenko et al. [33] using publicly available HGSOC Visium data (GSE211956, 8 patient samples: P1–P8). (**a**) Tumor cell fraction spatial maps from RCTD deconvolution across all patients. The publicly deposited scRNA-seq reference contains 8 consolidated cell types (the original study used a 12-type annotation with 5 fibroblast subtypes; see Methods). Color intensity indicates tumor fraction (yellow = low, red = high). Tumor-enriched spots (RCTD tumor weight ≥ 0.15) were identified for downstream analysis. (**b**) inferCNV-based copy number cluster mapping reveals spatially distinct CNV profiles across patients. Multiple CNV clusters (3–6 per patient) were identified in all 8 samples via Leiden clustering on the inferred CNV matrix, consistent with the original study’s observation of genomic heterogeneity validated by whole-genome sequencing. Note: the original study used manual dendrogram-based splitting; automated Leiden clustering may yield different partition granularities. (**c**) Cell type composition across patients from RCTD deconvolution, showing variable proportions of tumor cells, macrophages, fibroblasts, T cells, B/plasma cells, endothelial cells, mesothelial cells, and myofibroblasts. (**d**) Quantitative summary of tumor burden (vermillion bars, left axis: percentage of tumor-enriched spots) and genomic instability (blue bars, right axis: mean CNV score) across patients, enabling cross-patient comparison of microenvironment dynamics. (**e**) Spatially variable gene ranking for patient P3 by Moran’s *I*, with cell type marker genes (*CD14*, *KRT7*, *CDH5*; red) and CNV-cluster marker genes (blue) highlighted. Cell type markers rank in the top 2% of spatially variable genes, while CNV markers are dispersed across the distribution. (**f**) Comparison of Moran’s *I* distributions between CNV-cluster marker genes and background genes (Mann–Whitney *U* test, *p* = 0.07; not significant), indicating that spatial organization in P3 is shaped more by microenvironment composition than by CNV-cluster identity.

To demonstrate ChatSpatial’s capacity for complex, multi-sample analytical workflows, we replicated the core findings of a recent study by Denisenko et al. on high-grade serous ovarian carcinoma (HGSOC) [33]. The original study profiled eight patient samples spanning different chemotherapy response categories and used an intricate computational pipeline to identify tumor subclones and characterize their microenvironments. We reproduced this workflow on all eight patient samples (P1–P8) using ChatSpatial’s conversational interface (Figure 3).

##### Step 1: Multi-Sample Integration and Tumor Identification

The workflow began with processing all eight patient samples. We prompted: *“Load the HGSOC Visium samples from GSE211956 and deconvolve cell types using RCTD with the study’s scRNA-seq reference.”* This single instruction triggered batch processing across the cohort, applying RCTD deconvolution [34] with the study’s scRNA-seq reference (8 cell types in the publicly deposited version; the original study used a finer 12-type annotation that subdivided fibroblasts into 5 subtypes). The platform automatically identified tumor-enriched spots (RCTD tumor weight ≥ 0.15, following the original study’s convention) across all samples, enabling systematic downstream analysis (Figure 3a,c).

##### Step 2: Subclone Discovery via Copy Number Inference

Next, we identified candidate subclonal populations through copy number inference. We prompted: *“For each sample, run inferCNV using low-tumor spots as the normal reference to identify regions with distinct copy number alteration profiles.”* The platform executed inferCNV [35] per sample, using spots with low RCTD tumor weights (< 0.15) as the reference profile—a critical methodological detail from the original study. Leiden clustering on the inferred CNV matrix revealed multiple spatially distinct CNV clusters in all 8 patients (3–6 clusters per sample), consistent with the original study’s finding of pervasive copy number heterogeneity across the HGSOC cohort (Figure 3b).

##### Step 3: Cross-Patient Microenvironment Comparison

Finally, we compared tumor microenvironments across patients. We asked: *“Summarize the cell type composition and tumor burden across all patients. Which patients show the highest genomic instability?”* The resulting cross-patient analysis revealed substantial heterogeneity in both tumor burden (ranging from 2% to 98% tumor-enriched spots) and genomic instability (mean CNV scores 0.012–0.024), with patients harboring the highest tumor fractions (P3, P7) also exhibiting the most complex sub-clonal architectures (Figure 3c–d). These patterns are consistent with the original study’s observation that intra-tumor heterogeneity varies substantially across patients and may influence differential treatment response.

##### Beyond Replication: Cross-Method Spatial Analysis

To illustrate schema-enforced orchestration across analytical methods, we selected patient P3—the sample with the highest tumor burden (96% tumor-enriched spots) and four distinct CNV clusters—and used a single conversational prompt to perform spatial autocorrelation (Moran’s *I* [36]) across 2,134 expressed genes. Cell type markers from the RCTD deconvolution—*CD14* (monocyte/macrophage, rank 16/2,134), *KRT7* (epithelial/tumor, rank 24), and *CDH5* (vascular endothelial, rank 39)—emerged among the top 2% of spatially variable genes (Moran’s *I*, FDR-corrected *p <* 0.05 via Benjamini–Hochberg). In contrast, the 69 testable CNV-cluster marker genes (top 20 differentially expressed genes per cluster) showed no enrichment for spatial patterning relative to background (Mann–Whitney *U* test comparing Moran’s *I* distributions, *p* = 0.99; *n*_markers_ = 69, *n*_background_ = 2,065). This is a single-sample observation with limited biological inference power; it does not establish that microenvironment composition generally dominates CNV-driven spatial organization in HGSOC. Rather, it demonstrates the feasibility of chaining deconvolution, CNV inference, and spatial statistics through conversational prompts—an exploratory analysis not reported in the original study (Figure 3e–f).

Together, these two case studies demonstrate that ChatSpatial can orchestrate complex, multi-tool analytical workflows—spanning spatial domain discovery, deconvolution, copy number inference, cell communication, and cross-cohort comparison—through a conversational workflow, achieving concordance with expert-curated published findings. Both case studies also demonstrate the feasibility of cross-method exploratory analyses: spatial autocorrelation tests, performed through single follow-up prompts, connected results across independent analytical frameworks—hypothesis-generating investigations that warrant further validation.

### 2.3 Interactive Reasoning and Platform Robustness

Beyond the case studies above, we systematically evaluated ChatSpatial’s robustness across diverse analytical conditions and its reproducibility at the workflow level under controlled experimental settings.

To validate functional coverage across diverse analytical conditions, we evaluated the platform on 28 test scenarios covering four categories: data handling and preprocessing (5 scenarios), core spatial analysis (11 scenarios), conversational workflows (5 scenarios), and scalability stress tests (7 scenarios) (Supplementary Table S2). These scenarios spanned extreme data regimes—from low-density STARmap data (300 spots, 150 genes) to ultra-high-resolution Xenium datasets (930K+ cells)—as well as complex multi-sample integration (6 Visium slides requiring batch correction) and deliberate edge cases including invalid inputs, missing spatial coordinates, and ambiguous user commands. All 28 scenarios completed successfully through conversational interaction alone, without manual code intervention. In 3 of 28 scenarios involving ambiguous prompts (e.g., under-specified clustering resolution or generic “analyze the data” commands), the LLM’s initial parameter selection required one round of conversational refinement—demonstrating the value of the human-in-the-loop design over fully autonomous execution. We note that these 28 scenarios assess functional coverage rather than statistical reproducibility; the latter is addressed by the cross-model determinism experiment below.

Beyond functional validation, we assessed reproducibility at the workflow level through two complementary experiments. First, we conducted a cross-model determinism experiment. We presented eight representative analysis prompts—spanning spatial domain identification, deconvolution, SVG detection, cell communication, and CNV inference—to three LLMs (Gemini 2.5 Flash, Claude Haiku 4.5, GPT-5 Mini) with identical MCP tool schemas, repeating each prompt 10 times at temperature = 1.0 (maximum sampling stochasticity). We deliberately selected lighter-weight models for this experiment to test whether schema enforcement maintains consistency even with less capable LLMs; the case study analyses used the more capable Claude Sonnet 4.5. We measured three metrics: *tool selection consistency* (fraction of trials selecting the same tool as the mode), *constrained parameter consistency* (agreement rate on Literalenumerated parameters such as method names), and *free-text parameter consistency* (agreement rate on unconstrained string inputs). Tool selection was 100% consistent across all 240 trials—an expected result given that each prompt was designed to map unambiguously to a single tool category; testing with deliberately ambiguous prompts (e.g., “help me understand the tissue organization”) would likely reduce this consistency and represents a direction for future evaluation. Schema-constrained parameters showed 75.7% cross-model consistency, compared to 58.3% for free-text parameters, confirming that schema constraints substantially reduce—though do not eliminate—parameter variability across models. Per-model constrained parameter consistency ranged from 58.3% (GPT-5 Mini) to 100% (Claude Haiku 4.5), with Gemini 2.5 Flash at 68.8%. Full per-prompt breakdowns are provided in the reproducibility repository.

Second, to quantify the practical advantage of schema enforcement over free-form code generation, we presented the same eight prompts to the same three LLMs but asked them to write executable Python code instead of selecting from MCP tool schemas (240 total trials). To ensure a fair comparison, we report results separately for six Python-native tasks (spatial domains, SVG detection, Moran’s *I*, visualization, trajectory, CNV inference) and two cross-ecosystem tasks (RCTD deconvolution, CellChat communication) where code generation faces an inherent disadvantage because the requested tools are R-only. On Python-native tasks alone, the two models that reliably generated code exhibited substantial syntax error rates: Gemini 2.5 Flash at 41.7% (25/60 trials produced unparseable code) and Claude Haiku 4.5 at 15.0% (9/60). Error rates were highest for multi-step analyses requiring chained library calls—trajectory inference (90% syntax errors for Gemini, 40% for Claude) and spatial autocorrelation (90%, 10%)—while simpler single-function tasks (SVG detection, visualization) achieved near-perfect syntax. Models also hallucinated non-existent packages: Gemini invented pyrctd, pyCellChat,  and spacemk;  Claude attempted to import cellchat (an R-only package with no Python counterpart). GPT-5 Mini failed to produce any code for 5 of 8 tasks. On cross-ecosystem tasks, all models either hallucinated Python wrappers for R-only tools or produced code that bypassed the requested method entirely—a structural limitation that schema-enforced orchestration resolves by routing R-based methods through automated rpy2 bridges invisible to both the LLM and the user. We note that this comparison represents a lower bound for code generation approaches, as providing the LLMs with documentation or few-shot examples (as in RAG-augmented systems like STAgent) would likely reduce error rates. Our comparison isolates the effect of schema enforcement versus unconstrained generation.

Performance scaling was validated across datasets ranging from 1,000 to 45,000 spots and up to 930K+ cells, spanning seven major spatial transcriptomics technologies: SPOTS, Visium, MERFISH [37], seqFISH [38], STARmap [39], Slide-seq [40], and Xenium. All analyses completed without out-of-memory errors or timeouts; execution time is dominated by the underlying tool (e.g., seconds for clustering, minutes for deconvolution) with negligible overhead from the MCP orchestration layer. Dataset accession numbers and sources are provided in the Data Availability section.

## 3 Discussion

Our central result is that schema-enforced orchestration can coordinate complex, cross-ecosystem spatial transcriptomics workflows with high reproducibility at the workflow level while keeping the researcher in strategic control. Rather than repeating implementation details, we focus here on what the case studies and architectural evaluation reveal about the strengths, boundaries, and future directions of this approach.

The replication of two published studies—spanning HGSOC subclonal heterogeneity and OSCC tumor architecture (§2.2)—demonstrates that conversational orchestration can achieve concordance with expert-curated pipelines without sacrificing analytical rigor. Critically, these workflows each required crossing the Python/R divide (FlashDeconv deconvolution in Python followed by CellChat in R; RCTD in R followed by inferCNV in Python), a task that traditionally demands substantial scripting effort. That a conversational workflow can coordinate these cross-ecosystem transitions—while maintaining data integrity and methodological fidelity—represents a meaningful step toward closing the gap between biological questions and computational answers.

This design reflects a broader principle: scientific discovery is iterative, yet traditional analysis imposes a disjointed cycle of planning, coding, and debugging that slows exploration. By removing implementation friction between analytical steps, ChatSpatial allows researchers to pursue follow-up questions as they emerge—*“What are the marker genes for this cluster?”* followed by *“Is there enrichment of any signaling pathway?”*—maintaining the natural rhythm of hypothesis-driven inquiry while the LLM handles technical execution.

A key consequence of low-friction tool chaining is that exploratory analyses become routine rather than exceptional. The “Beyond Replication” results in both case studies (§2.2) illustrate this: in each, a single follow-up prompt connected findings from one analytical framework to an independent spatial statistics method. These cross-method analyses were not planned *a priori* but emerged naturally from the conversational workflow—precisely the kind of exploratory analyses that implementation overhead might otherwise discourage.

ChatSpatial occupies a distinct position in the computational biology landscape through its philosophy of human-steered orchestration (Supplementary Table S3). Unlike fully autonomous agents such as STAgent [12] and SpatialAgent [13] that aim to replace the research workflow with minimal human input, ChatSpatial prioritizes reliability and reproducibility through schema-enforced execution via MCP. This design choice is supported by recent findings that current single-cell LLMs do not necessarily outperform traditional, task-specific methods across all benchmarks [6]. Our approach leverages the proven strengths of the existing ecosystem—orchestrating established, best-in-class tools—rather than attempting to replace them. Furthermore, unlike agents operating within a single programming ecosystem, ChatSpatial provides a unified interface over the fragmented R and Python environments, enabling cross-platform analytical strategies previously impractical due to language and toolchain fragmentation. Tools like CellWhisperer, which leverage learned multimodal embeddings for zero-shot semantic exploration, address a complementary challenge; we envision future integration where such models could operate as tools within ChatSpatial’s orchestration framework.

We also note that our comparison in Table 1 focuses on programmatic workflows. GUI-based tools such as 10x Genomics Loupe Browser, Giotto Viewer, and web platforms like Galaxy offer accessible alternatives for specific analytical tasks. However, these tools typically cover a narrower analytical scope and do not support the kind of cross-ecosystem, multi-method chaining that ChatSpatial enables. The approaches are complementary: GUI tools excel at interactive visualization and targeted exploration, while ChatSpatial addresses the integration challenge across dozens of specialized methods spanning Python and R.

Despite these strengths, ChatSpatial has several limitations that define key areas for future work. First, the platform’s performance is fundamentally linked to its core components: the LLM’s reasoning ability and the underlying bioinformatics tools. The natural language interface, while powerful, can misinterpret ambiguous queries, and its parameter guidance currently relies on manually curated documentation. While our MCP-based architecture ensures the platform is LLM-agnostic and not locked into any single model provider, automated parameter optimization remains an exploratory direction that would require rigorous validation to ensure reliable quantitative exploration beyond curated defaults.

Second, while ChatSpatial eliminates cross-ecosystem integration complexity, its current command-line interface still requires familiarity with terminal environments and SSH connections. This positions the platform primarily for researchers with some computational background—domain experts comfortable with command-line tools and computational biologists seeking to accelerate their workflows. To extend accessibility to bench biologists and clinicians who generate spatial data but lack computational training, our immediate roadmap prioritizes the development of an open-source, web-based user interface. This browser-based deployment will remove the final technical barrier, fulfilling our broader goal of democratizing advanced spatial analysis.

Third, the conversational paradigm introduces practical trade-offs: LLM API calls add latency and cost compared to direct scripting, and sending dataset descriptions to external LLM providers raises data privacy considerations for sensitive clinical datasets. Local model deployment could mitigate privacy concerns, though at a potential cost to reasoning quality.

Fourth, our case study validation relies on replication of published analyses, and we cannot exclude the possibility that the LLM’s parameter choices were partly informed by exposure to these studies during pretraining. While the schema constraints limit the parameter space regardless of the model’s prior knowledge, a blinded evaluation on unpublished datasets would provide stronger evidence of generalizability and remains an important direction for future work.

Fifth, while ChatSpatial integrates 60+ methods, tool selection inevitably reflects curatorial judgment. Notable methods not yet integrated (e.g., BayesSpace, Giotto) may be preferred for specific applications, and our inclusion criteria—benchmark-validated performance, community adoption, and cross-platform coverage—may not capture all emerging methods.

Sixth, although the schema-enforced architecture simplifies tool updates relative to code-generation approaches, maintaining compatibility with evolving upstream packages (e.g., API changes across major scanpy versions) requires ongoing wrapper maintenance—a practical consideration for long-term sustainability.

Finally, as an integration platform, the scientific validity of ChatSpatial’s results ultimately depends on the quality and correctness of the community-developed tools it integrates. While our schema-enforced architecture mitigates errors from LLM hallucinations, the platform inherits the intrinsic strengths and weaknesses of the methods in its toolkit.

The growing complexity of spatial transcriptomics analysis has created a bottleneck, limiting discovery to computationally specialized labs. ChatSpatial addresses this by shifting the paradigm from tool-centric programming to science-centric conversation, using schema-enforced orchestration across the Python/R divide to make complex comparative analyses routine. By aligning computational analysis with the natural rhythm of scientific inquiry, ChatSpatial takes a step toward a future where discovery is driven by the depth of biological questions, not the mastery of code.

## 4 Methods

### 4.1 Architecture: Schema-Enforced Reproducibility via the Model Context Protocol

To address the critical challenge of computational reproducibility in LLM-driven science, ChatSpatial is built upon the Model Context Protocol (MCP) [17], an open standard developed by Anthropic that is increasingly being adopted for integrating complex scientific software. Unlike traditional agents that rely on LLMs to generate free-form code—a stochastic process prone to hallucinating non-existent functions or producing unparseable syntax (15–42% syntax error rates even on Python-native tasks in our baseline experiment, §2.3)—our architecture imposes a strict **schema-enforced execution model**.

Conceptually, this shifts the LLM’s role from writing a “free-form essay” (code generation) to solving a “fill-in-the-blank” problem (parameter selection). We expose 60+ bioinformatics methods through 20 high-level MCP tools, where each tool represents an analytical category (e.g., deconvolve data, identify spatial domains) and accepts a method parameter that routes to specific algorithm implementations. These tools are not open-ended prompts but rigidly defined interfaces with strict input/output schemas. For instance, when a user requests deconvolution, the LLM cannot invent a method name; it must select from a pre-validated enumeration (e.g., Literal[“rctd”, “card”, “cell2location”]). This constraint mechanism effectively decouples the probabilistic nature of the LLM’s reasoning (understanding intent) from the deterministic nature of the tool’s execution (running the algorithm). Quantitatively, across the 20 MCP tools encompassing 441 total parameters, 358 (81.2%) are schema-constrained through Literal enumerations, numeric bounds, or default values; only 83 parameters (18.8%) accept free-text input, primarily dataset identifiers and column names that reference existing data structures rather than open-ended specifications.

By enforcing type safety and parameter validity *before* any code is executed, the MCP architecture eliminates common failure modes such as syntax errors and package incompatibilities. For fixed tool choices and parameter values, execution follows deterministic software paths under fixed package and environment versions; residual variability therefore resides mainly in the LLM’s intent-to-parameter mapping, which schema constraints substantially narrow (81.2% of parameters are enumerated or bounded). In a cross-model determinism experiment (§2.3), tool selection was 100% consistent across 240 trials at temperature = 1.0, with schema-constrained parameters showing 75.7% consistency versus 58.3% for free-text inputs. The protocol is increasingly being adopted as an industry standard, with 10x Genomics [41] and Benchling [42] releasing official MCP integrations in 2025. Our implementation supports the 2025–03-26 MCP specification through the Python SDK (v1.17.0+). Further details on the protocol architecture and system prompts are provided in the Supplementary Methods (Section S1).

### 4.2 Integration Architecture and Method Abstraction

We integrated the full method library (Supplementary Table S1) through an MCP-based tool integration architecture designed to ensure both analytical fidelity and a consistent interface for the LLM agent. The architecture consists of three operational layers:

1. **MCP Tool Layer.** Each analysis function is registered as an MCP tool via the @mcp.tool() decorator, exposing it to the LLM with strict input/output schemas that enforce parameter validation and type safety.
2. **Method Dispatch Layer.** Registered tools perform data validation, coordinate management, and route requests to algorithm-specific implementations based on userspecified method parameters.
3. **Algorithm Wrapper Layer.** Method-specific wrappers handle data preparation (coordinate extraction, quality checks, format conversions), call the original software with appropriate parameters, and standardize outputs into consistent result objects.

For methods that are only available in R—such as CellChat for cell communication analysis—the wrapper layer utilizes rpy2 to call R from within the Python-based server, automatically converting AnnData objects to Seurat objects and translating results back. This inter-language process is encapsulated within the wrapper and invisible to the user. A detailed example of this integration process for SpaGCN spatial domain identification is provided in the Supplementary Methods (Section S2).

### 4.3 Knowledge Injection via Protocol: Documentation-Driven Parameter Inference

Instead of relying on rigid rule-based engines or resource-intensive model fine-tuning, ChatSpatial implements a systematic “Knowledge Injection via Protocol” strategy. We embed expert biological knowledge directly into the MCP tool schemas, transforming the interface from a passive validation layer into an active guide for the LLM.

Specifically, every parameter field in our schema includes not only data type constraints but also rich, context-aware semantic descriptions accessible to the LLM. For instance, in the spatial graph construction tool (build spatial graph), the description for the n neighbors parameter explicitly encodes technology-specific heuristics: *“For 10x Visium (hexagonal grid), recommended k=6 for immediate neighbors; for single-cell resolution platforms like MERFISH, recommended k=10–15 based on cell density; for Xenium, consider Delaunay triangulation for natural cell neighborhoods.”* Similarly, the clustering resolution parameter documentation embeds biological guidance: *“Higher values (1.5–2.0) produce more numerous, smaller clusters ideal for detecting rare cell types; lower values (0.2–0.5) produce fewer, broader clusters suitable for identifying major tissue domains.”* These descriptions encode years of methodological benchmarking and biological expertise in a format directly interpretable by the LLM.

When the LLM processes a user request (e.g., “Analyze this MERFISH dataset”), it performs a three-step **in-context inference** process: (1) it *retrieves* the relevant tool schema from the MCP registry, (2) it *reads* the embedded parameter documentation to understand platform-specific requirements, and (3) it *infers* the optimal parameter values based on the dataset context. For example, upon detecting MERFISH data characteristics (high spatial resolution, single-cell precision), the LLM dynamically selects n neighbors=10 without hallucinating arbitrary values. This approach decouples domain knowledge from the model weights, allowing for a **lightweight and extensible** system where methodological best practices can be updated simply by modifying the schema descriptions—eliminating the need for costly model retraining when new spatial technologies emerge (e.g., Visium HD, CosMx). Further examples of our knowledge-rich schema documentation are provided in the Supplementary Methods (Section S3).

### 4.4 Error Handling and Recovery Mechanisms

ChatSpatial implements error handling designed for conversational interaction rather than silent recovery. The core design principle is that errors are converted into structured result objects and returned to the LLM, enabling it to observe failures and guide users toward solutions through natural language rather than requiring manual debugging. This is achieved through two key mechanisms: (1) a semantic exception hierarchy that categorizes errors by type (data issues, parameter errors, processing failures, dependency problems), enabling the LLM to provide targeted guidance; and (2) the @mcp_tool_error_handler decorator that wraps all MCP tools with type-aware error responses, maintaining protocol-level consistency while returning actionable diagnostic messages. Detailed implementation is provided in the Supplementary Methods (Section S4).

## Supplementary Material

Supplement 1

Supplementary Information

Supplementary information is available for this paper. This includes Supplementary Methods, Supplementary Tables, and detailed documentation of integrated methods.

## Figures and Tables

**Table 1:** Comparison of analysis paradigms for spatial transcriptomics: conversational orchestration, GUI-based platforms, and direct programming.

Workflow Aspect	ChatSpatial (Conversational Orchestration)	GUI Platforms (e.g., Loupe Browser, Giotto Viewer)	Direct Programming (Python/R)

**Primary Unit of Interaction**	A high-level scientific goal (e.g., “Find spatial domains”)	A menu-driven selection or button click (e.g., select clustering algorithm)	A specific line of code (e.g., sc.tl.leiden(...))
**Cognitive Focus of User**	Biological hypothesis and interpretation of results	Navigation of software interface and understanding of available options	Correct syntax, library versions, and data object manipulation
**Handling Multi-Step Analysis**	Fluid conversation where context is maintained by the agent	Limited to workflows supported by the platform; multi-tool chaining often unavailable	Manual scripting, requiring the user to manage state and data flow
**Cross-Language Integration (e.g., Python to R)**	Automated and hidden from the user. The agent manages data conversion internally.	Generally confined to a single ecosystem (Python or R); interoperability not supported	Manual and errorprone. Requires writing explicit export/import scripts (e.g., via zellkonverter).
**Parameter Selection**	Agent suggests best practices based on dialogue and data context	Exposed through interface controls with platform-set defaults	User must manually look up and specify parameters in code
**Source of Reproducibility**	The immutable log of the scientific conversation	Session logs or exported configuration files, where available	User-maintained script files and environment configurations (requirements.txt, renv.lock)
**Role of Foundational Libraries (Scanpy, Seurat, etc.)**	Act as reliable, versioned execution engines orchestrated by the agent.	Bundled internally; user interacts through the GUI layer	Act as the direct, code-level interface for the user.

**Table 2:** Platform compatibility matrix: analytical module support across spatial transcriptomics technologies. **Legend:** ✓✓= Optimized/Recommended, ✓= Supported, ° = Optional, − = Not Required/Not Applicable. Cell-resolution technologies (MERFISH, Xenium, seqFISH, CosMx) do not require deconvolution as they provide native single-cell data. Spot-based platforms (Visium, Slide-seq) benefit from deconvolution to infer cell-type compositions. All modules support standard H5AD format with optional spatial coordinates stored in adata.obsm[’spatial’].

Module	Visium	Visium HD	MERFISH Xenium	seqFISH CosMx	Slide-seq	STAR map

**Preprocessing**	✓	✓	✓	✓	✓	✓
**Multi-Sample Integration**	✓	✓	✓	✓	✓	✓
**Cell Type Annotation**	✓	✓	✓	✓	✓	✓
**Differential Expression**	✓	✓	✓	✓	✓	✓
**Spatial Domain ID**	✓✓	✓	✓	✓	✓	✓
**Spatial Variable Genes**	✓	✓	✓	✓	✓	✓
**Cell Communication**	✓	✓	✓✓	✓✓	✓	✓
**Deconvolution**	✓✓	°	−	−	°	°
**CNV Analysis**	✓	✓	✓	✓	✓	✓
**Trajectory Analysis**	✓	✓	✓✓	✓✓	✓	✓
**RNA Velocity**	✓	✓	✓	✓	✓	✓
**Enrichment Analysis**	✓	✓	✓	✓	✓	✓

## Data Availability

All public datasets used for benchmarking and validation in this study are listed below and documented in a dedicated reproducibility repository (https://github.com/cafferychen777/ChatSpatial-Reproducibility): • Oral Squamous Cell Carcinoma (OSCC) Visium dataset: GEO accession GSE208253 [32] • High-Grade Serous Ovarian Carcinoma (HGSOC) Visium dataset: GEO accession GSE211956 [33] • HNSCC single-cell reference: GEO accession GSE103322 [31] Additional datasets used for platform validation (Section 2.3) include SPOTS (GSE198353), Visium multi-sample benchmarks (GSE254652, GSE243275), MERFISH (GSE113576), seqFISH (GSE133244), STARmap (https://www.wangxiaolab.org/data-portal-1), Slide-seq (Broad Institute Single Cell Portal SCP354), and Xenium (10x Genomics public dataset). All datasets are publicly available, and no custom datasets were generated for this study.

## References

[1] Rao A., Barkley D., França G.S., Yanai I.: Exploring tissue architecture using spatial transcriptomics. Nature 596(7871), 211–220 (2021) 10.1038/s41586-021-03634-9 34381231 PMC8475179

[2] Chen K.H., Boettiger A.N., Moffitt J.R., Wang S., Zhuang X.: Spatially resolved, highly multiplexed rna profiling in single cells. Science 348(6233), 6090 (2015) 10.1126/science.aaa6090 PMC4662681 25858977

[3] Moses L., Pachter L.: Museum of spatial transcriptomics. Nat. Methods 19(5), 534–546 (2022) 10.1038/s41592-022-01409-2 35273392

[4] Virshup I., Rybakov S., Theis F.J., al.: anndata: Annotated data. bioRxiv (2021) 10.1101/2021.12.16.473007

[5] Hao Y., Hao S., Andersen-Nissen E., al.: Integrated analysis of multimodal single-cell data. Cell 184(13), 3573–3587 (2021) 10.1016/j.cell.2021.04.048 34062119 PMC8238499

[6] Xie F., Zhao B., Xu S., Wang Z., Moon J.J., Garmire L.X.: Overcoming barriers to the wide adoption of single-cell large language models in biomedical research. Nat. Biotechnol. 43(11), 1758–1762 (2025) 10.1038/s41587-025-02846-y . Comment 41131148 PMC13520874

[7] Atta L., Fan J.: Computational challenges and opportunities in spatially resolved transcriptomic data analysis. Nat. Commun. 12(1), 5283 (2021) 10.1038/s41467-021-25557-9 34489425 PMC8421472

[8] Dries R., Zhu Q., Dong R., Eng C.-H.L., Li H., Liu K., Fu Y., Zhao T., Sarkar A., Bao F., George R.E., Pierson N., Cai L., Yuan G.-C.: Giotto: a toolbox for integrative analysis and visualization of spatial expression data. Genome Biol. 22(1), 78 (2021) 10.1186/s13059-021-02286-2 33685491 PMC7938609

[9] Huang C.H.: Qust-llm: Integrating large language models for comprehensive spatial transcriptomics analysis (2024) 10.48550/arXiv.2406.14307arXiv:2406.14307 [q-bio.GN]

[10] Nagarajan V., Shi G., Arunkumar S., Liu C., Gopalakrishnan J., Nath P.R., Jang J., Caspi R.R.: SCassist: An AI Based Workflow Assistant for Single-Cell Analysis. Bioinformatics 41(8), 402 (2025) 10.1093/bioinformatics/btaf402 PMC12341677 40650988

[11] Schaefer M., Peneder P., Malzl D., Lombardo S.D., Peycheva M., Burton J., Hakobyan A., Sharma V., Krausgruber T., Sin C., Menche J., Tomazou E.M., Bock C.: Multimodal learning enables chat-based exploration of single-cell data. Nat. Biotechnol. (2025) 10.1038/s41587-025-02857-9 PMC13654808 41219484

[12] Lin Z., Wang W., Marin-Llobet A., Li Q., Pollock S.D., Sui X., Aljovic A., Lee J., Baek J., Liang N., Zhang X., Wang C.K., Huang J., Liu M., Gao Z., Sheng H., Du J., Lee S.J., Wang B., He Y., Ding J., Wang X., Alvarez-Dominguez J.R., Liu J.: Spatial transcriptomics ai agent charts hpsc-pancreas maturation in vivo (2025) 10.1101/2025.04.01.646731bioRxiv:2025.04.01.646731

[13] Wang H., He Y., Coelho P.P., Bucci M., Nazir A., Chen B., Trinh L., Zhang S., Huang K., Chandrasekar V., Chung D.C., Hao M., Leote A.C., Lee Y., Li B., Liu T., Liu J., Lopez R., Lucas T., Ma M., Makarov N., McGinnis L., Peng L., Ra S., Scalia G., Singh A., Tao L., Uehara M., Wang C., Wei R., Copping R., Rozenblatt-Rosen O., Leskovec J., Regev A.: Spatialagent: An autonomous ai agent for spatial biology. bioRxiv (2025) 10.1101/2025.04.03.646459

[14] Spracklen J., Wijewickrama R., Sakib A.H.M.N., Maiti A., Viswanath B., Jadliwala M.: We have a package for you! a comprehensive analysis of package hallucinations by code generating llms. arXiv preprint arXiv:2406.10279 (2024). To appear in USENIX Security 2025

[15] Wang Z., Zhou Z., Song D., Huang Y., Chen S., Ma L., Zhang T.: Towards understanding the characteristics of code generation errors made by large language models. arXiv preprint arXiv:2406.08731 (2024). To appear in ICSE 2025

[16] Dinh T., Zhao J., Tan S., Negrinho R., Lausen L., Zha S., Karypis G.: Large language models of code fail at completing code with potential bugs. In: Advances in Neural Information Processing Systems, vol. 36, pp. 41386–41412. Curran Associates, Inc., Red Hook, NY (2023)

[17] Anthropic: Model Context Protocol. Accessed: 2025-10-30 (2024). https://modelcontextprotocol.io/

[18] Anthropic: Claude 3.5 Sonnet. Accessed: 2025-10-30 (2024). https://www.anthropic.com/news/claude-3-5-sonnet

[19] Tian L., Chen F., Macosko E.Z.: The expanding vistas of spatial transcriptomics. Nat. Biotechnol. 41(6), 773–782 (2023) 10.1038/s41587-022-01448-2 36192637 PMC10091579

[20] Li H., Zhou J., Li Z., Chen S., Liao X., Zhang B., Zhang R., Wang Y., Sun S., Gao X.: A comprehensive benchmarking with practical guidelines for cellular deconvolution of spatial transcriptomics. Nat. Commun. 14(1), 1548 (2023) 10.1038/s41467-023-37168-7 36941264 PMC10027878

[21] Kang L., Zhang Q., Qian F., Liang J., Wu X.: Benchmarking computational methods for detecting spatial domains and domain-specific spatially variable genes from spatial transcriptomics data. Nucleic Acids Res. 53(7), 303 (2025) 10.1093/nar/gkaf303 PMC12000868 40240000

[22] Hu Y., Xie M., Li Y., Rao M., Shen W., Luo C., Qin H., Baek J., Zhou X.M.: Benchmarking clustering, alignment, and integration methods for spatial transcriptomics. Genome Biol. 25(1), 212 (2024) 10.1186/s13059-024-03361-0 39123269 PMC11312151

[23] Sang-aram C., Browaeys R., Seurinck R., Saeys Y.: Spotless, a reproducible pipeline for benchmarking cell type deconvolution in spatial transcriptomics. eLife 12, 88431 (2024) 10.7554/eLife.88431 PMC11126312 38787371

[24] Li Z., Patel Z.M., Song D., Yasa S.N., Cannoodt R., Yan G., Li J.J., Pinello L.: Systematic benchmarking of computational methods to identify spatially variable genes. Genome Biol. 26(1), 285 (2025) 10.1186/s13059-025-03731-2 40968359 PMC12445034

[25] Luo Y., Ren J., Yang Q., Zhou Y., You Z., Li Q.: Benchmarking rna velocity methods across 17 independent studies. bioRxiv (2025) 10.1101/2025.08.02.668272 PMC13106975 41916302

[26] Dimitrov D., Schäfer P.S.L., Farr E., Rodriguez-Mier P., Lobentanzer S., Badia-i-Mompel P., Dugourd A., Tanevski J., Ramirez Flores R.O., SaezRodriguez J.: Liana+ provides an all-in-one framework for cell-cell communication inference. Nat. Cell Biol. 26, 1613–1622 (2024) 10.1038/s41556-024-01469-w 39223377 PMC11392821

[27] Jin S., Plikus M.V., Nie Q.: Cellchat for systematic analysis of cell-cell communication from single-cell transcriptomics. Nat. Protoc. 20, 180–219 (2025) 10.1038/s41596-024-01045-4 39289562

[28] Weiler P., Lange M., Klein M., Pe’er D., Theis F.: Cellrank 2: unified fate mapping in multiview single-cell data. Nat. Methods 21, 1196–1205 (2024) 10.1038/s41592-024-02303-9 38871986 PMC11239496

[29] Korsunsky I., Millard N., Fan J., Slowikowski K., Zhang F., Wei K., Baglaenko Y., Brenner M., Loh P. r., Raychaudhuri S.: Fast, sensitive and accurate integration of single-cell data with harmony. Nat. Methods 16(12), 1289–1296 (2019) 10.1038/s41592-019-0619-0 31740819 PMC6884693

[30] Lopez R., Regier J., Cole M.B., Jordan M.I., Yosef N.: Deep generative modeling for single-cell transcriptomics. Nat. Methods 15(12), 1053–1058 (2018) 10.1038/s41592-018-0229-2 30504886 PMC6289068

[31] Puram S.V., Tirosh I., Parikh A.S., Patel A.P., Yizhak K., Gillespie S., Rodman C., Luo C.L., Mroz E.A., Emerick K.S., : Single-cell transcriptomic analysis of primary and metastatic tumor ecosystems in head and neck cancer. Cell 171(7), 1611–1624 (2017) 10.1016/j.cell.2017.10.044 29198524 PMC5878932

[32] Arora R., Cao C., Kumar M., Sinha S., Chanda A., McNeil R., Samuel D., Arora R.K., Matthews T.W., Chandarana S., Hart R., Dort J.C., Biernaskie J., Neri P., Hyrcza M.D., Bose P.: Spatial transcriptomics reveals distinct and conserved tumor core and edge architectures that predict survival and targeted therapy response. Nat. Commun. 14, 5029 (2023) 10.1038/s41467-023-40271-4 37596273 PMC10439131

[33] Denisenko E., Kock L., Tan A., : Spatial transcriptomics reveals discrete tumour microenvironments and autocrine loops within ovarian cancer subclones. Nat. Commun. 15, 2860 (2024) 10.1038/s41467-024-47271-y 38570491 PMC10991508

[34] Cable D.M., Murray E., Zou L.S., al.: Robust decomposition of cell type mixtures in spatial transcriptomics. Nat. Biotechnol. 40(4), 517–526 (2022) 10.1038/s41587-021-00830-w 33603203 PMC8606190

[35] Patel A.P., Tirosh I., Trombetta J.J., Shalek A.K., Gillespie S.M., Wakimoto H., Cahill D.P., Nahed B.V., Curry W.T., Martuza R.L., Louis D.N., Rozenblatt-Rosen O., Suvà M.L., Regev A., Bernstein B.E.: Single-cell rna-seq highlights intratumoral heterogeneity in primary glioblastoma. Science 344(6190), 1396–1401 (2014) 10.1126/science.1254257 24925914 PMC4123637

[36] Palla G., Spitzer H., Klein M., al.: Squidpy: a scalable framework for spatial omics analysis. Nat. Methods 19(2), 171–178 (2022) 10.1038/s41592-021-01358-2 35102346 PMC8828470

[37] Moffitt J.R., Bambah-Mukku D., Eichhorn S.W., al.: Molecular, spatial, and functional single-cell profiling of the hypothalamic preoptic region. Science 362(6416), 5324 (2018) 10.1126/science.aau5324 PMC6482113 30385464

[38] Lohoff T., Ghazanfar S., Missarova A., al.: Integration of spatial and single-cell transcriptomic data elucidates mouse organogenesis. Nat. Biotechnol. 40(1), 74–85 (2022) 10.1038/s41587-021-01006-2 34489600 PMC8763645

[39] Wang X., Allen W.E., Wright M.A., al.: Three-dimensional intact-tissue sequencing of single-cell transcriptional states. Science 361(6400), 5691 (2018) 10.1126/science.aat5691 PMC6339868 29930089

[40] Rodriques S.G., Stickels R.R., Goeva A., al.: Slide-seq: A scalable technology for measuring genome-wide expression at high spatial resolution. Science 363(6434), 1463–1467 (2019) 10.1126/science.aaw1219 30923225 PMC6927209

[41] 10x Genomics: Prompt-Based Interface for the 10x Genomics Cloud. Accessed: 2025-10-30 (2025). https://www.10xgenomics.com/support/software/cloud-analysis/latest/tutorials/cloud-mcp-server

[42] Benchling: Benchling MCP. Accessed: 2025-10-30 (2025). https://help.benchling.com/hc/en-us/articles/40342713479437-Benchling-MCP

[43] Wolf F.A., Angerer P., Theis F.J.: Scanpy: large-scale single-cell gene expression data analysis. Genome Biol. 19(1), 15 (2018) 10.1186/s13059-017-1382-0 29409532 PMC5802054

[44] Marconato L., Palla G., Yamauchi K.A., al.: Spatialdata: an open and universal data framework for spatial omics. Nat. Methods 22(1), 58–62 (2025) 10.1038/s41592-024-02212-x 38509327 PMC11725494

[45] Hafemeister C., Satija R.: Normalization and variance stabilization of single-cell rna-seq data using regularized negative binomial regression. Genome Biol. 20(1), 296 (2019) 10.1186/s13059-019-1874-1 31870423 PMC6927181

[46] Polański K., Young M.D., Miao Z., Meyer K.B., Teichmann S.A., Park J.-E.: Bbknn: fast batch alignment of single cell transcriptomes. Bioinformatics 36(3), 964–965 (2020) 10.1093/bioinformatics/btz625 31400197 PMC9883685

[47] Hie B., Bryson B., Berger B.: Efficient integration of heterogeneous single-cell transcriptomes using scanorama. Nat. Biotechnol. 37(6), 685–691 (2019) 10.1038/s41587-019-0113-3 31061482 PMC6551256

[48] Zeira R., Land M., Strzalkowski A., Raphael B.J.: Alignment and integration of spatial transcriptomics data. Nat. Methods 19(5), 567–575 (2022) 10.1038/s41592-022-01459-6 35577957 PMC9334025

[49] Clifton K., Anant M., Aihara G., Atta L., Aimiuwu O.K., Kebschull J.M., Miller M.I., Tward D., Fan J.: Stalign: Alignment of spatial transcriptomics data using diffeomorphic metric mapping. Nat. Commun. 14(1), 8123 (2023) 10.1038/s41467-023-43915-7 38065970 PMC10709594

[50] Hunter J.D.: Matplotlib: A 2d graphics environment. Comput. Sci. Eng. 9(3), 90–95 (2007) 10.1109/MCSE.2007.55

[51] Biancalani T., Scalia G., Buffoni L., al.: Deep learning and alignment of spatially resolved single-cell transcriptomes with tangram. Nat. Methods 18(11), 1352–1362 (2021) 10.1038/s41592-021-01264-7 34711971 PMC8566243

[52] Xu C., Lopez R., Mehlman E., Regier J., Jordan M.I., Yosef N.: Probabilistic harmonization and annotation of single-cell transcriptomics data with deep generative models. Mol. Syst. Biol. 17(1), 9620 (2021) 10.15252/msb.20209620 PMC7829634 33491336

[53] Zhang A.W., O’Flanagan C., Chavez E.A., al.: Probabilistic cell-type assignment of single-cell rna-seq for tumor microenvironment profiling. Nat. Methods 16(10), 1007–1015 (2019) 10.1038/s41592-019-0529-1 31501550 PMC7485597

[54] Yang C., Zhang X., Chen J.: Large language model consensus substantially improves the cell type annotation accuracy for scrna-seq data. bioRxiv (2025) 10.1101/2025.04.10.647852 42260142

[55] Ianevski A., Giri A.K., Aittokallio T.: Fully-automated and ultra-fast cell-type identification using specific marker combinations from single-cell transcriptomic data. Nat. Commun. 13(1), 1246 (2022) 10.1038/s41467-022-28803-w 35273156 PMC8913782

[56] Aran D., Looney A.P., Liu L., Wu E., Fong V., Hsu A., Chak S., Naikawadi R.P., Wolters P.J., Abate A.R., Butte A.J., Bhattacharya M.: Reference-based analysis of lung single-cell sequencing reveals a transitional profibrotic macrophage. Nat. Immunol. 20(2), 163–172 (2019) 10.1038/s41590-018-0276-y 30643263 PMC6340744

[57] Hu J., Li X., Coleman K., Schroeder A., Ma N., Irwin D.J., Lee E.B., Shinohara R.T., Li M.: Spagcn: Integrating gene expression, spatial location and histology to identify spatial domains and spatially variable genes by graph convolutional network. Nat. Methods 18(11), 1342–1351 (2021) 10.1038/s41592-021-01255-8 34711970

[58] Dong K., Zhang S.: Deciphering spatial domains from spatially resolved transcriptomics with an adaptive graph attention auto-encoder. Nat. Commun. 13(1), 1739 (2022) 10.1038/s41467-022-29439-6 35365632 PMC8976049

[59] Long Y., Ang K.S., Li M., al.: Spatially informed clustering, integration, and deconvolution of spatial transcriptomics with graphst. Nat. Commun. 14(1), 1155 (2023) 10.1038/s41467-023-36796-3 36859400 PMC9977836

[60] Traag V., Waltman L., Eck N.: From louvain to leiden: guaranteeing well-connected communities. Sci. Rep. 9(1), 5233 (2019) 10.1038/s41598-019-41695-z 30914743 PMC6435756

[61] Blondel V.D., Guillaume J.-L., Lambiotte R., Lefebvre E.: Fast unfolding of communities in large networks. J. Stat. Mech. 2008(10), 10008 (2008) 10.1088/1742-5468/2008/10/P10008

[62] Sun S., Zhu J., Zhou X.: Statistical analysis of spatial expression patterns for spatially resolved transcriptomic studies. Nat. Methods 17(2), 193–200 (2020) 10.1038/s41592-019-0701-7 31988518 PMC7233129

[63] Svensson V., Teichmann S.A., Stegle O.: Spatialde: identification of spatially variable genes. Nat. Methods 15(5), 343–346 (2018) 10.1038/nmeth.4636 29553579 PMC6350895

[64] Dimitrov D., Türei D., Garrido-Rodriguez M., al.: Comparison of methods and resources for cell-cell communication inference from single-cell rna-seq data. Nat. Commun. 13(1), 3224 (2022) 10.1038/s41467-022-30755-0 35680885 PMC9184522

[65] Efremova M., Vento-Tormo M., Teichmann S.A., Vento-Tormo R.: Cell-phonedb: inferring cell–cell communication from combined expression of multi-subunit ligand–receptor complexes. Nat. Protoc. 15(4), 1484–1506 (2020) 10.1038/s41596-020-0292-x 32103204

[66] Jin S., Guerrero-Juarez C.F., Zhang L., al.: Inference and analysis of cell-cell communication using cellchat. Nat. Commun. 12(1), 1088 (2021) 10.1038/s41467-021-21246-9 33597522 PMC7889871

[67] Hou S., Ma W., Zhou X.: Fastccc: a permutation-free framework for scalable, robust, and reference-based cell-cell communication analysis in single cell transcriptomics studies. Nat. Commun. 16, 66272 (2025) 10.1038/s41467-025-66272-z PMC12749929 41390348

[68] Yang C., Zhang X., Chen J.: Flashdeconv enables atlas-scale, multi-resolution spatial deconvolution via structure-preserving sketching. bioRxiv (2025) 10.64898/2025.12.22.696108 . Preprint

[69] Kleshchevnikov V., Shmatko A., Dann E., al.: Cell2location maps fine-grained cell types in spatial transcriptomics. Nat. Biotechnol. 40(5), 661–671 (2022) 10.1038/s41587-021-01139-4 35027729

[70] Elosua-Bayes M., Nieto P., Mereu E., Gut I., Heyn H.: Spotlight: seeded nmf regression to deconvolute spatial transcriptomics spots with single-cell transcriptomes. Nucleic Acids Res. 49(9), 50 (2021) 10.1093/nar/gkab043 PMC8136778 33544846

[71] Andersson A., Bergenstråhle J., Asp M., Bergenstråhle L., Jurek A., Fernández Navarro J., Lundeberg J.: Single-cell and spatial transcriptomics enables probabilistic inference of cell type topography. Commun. Biol. 3(1), 565 (2020) 10.1038/s42003-020-01247-y 33037292 PMC7547664

[72] Lopez R., Li B., Keren-Shaul H., Boyeau P., Kedmi M., Pilzer D., Jelinski A., Yofe I., David E., Wagner A., Ergen C., Addad Y., Golani O., Ronchese F., Jordan M.I., Amit I., Yosef N.: Destvi identifies continuums of cell types in spatial transcriptomics data. Nat. Biotechnol. 40(9), 1360–1369 (2022) 10.1038/s41587-022-01272-8 35449415 PMC9756396

[73] Ma Y., Zhou X.: Spatially informed cell-type deconvolution for spatial transcriptomics. Nat. Biotechnol. 40(9), 1349–1359 (2022) 10.1038/s41587-022-01273-7 35501392 PMC9464662

[74] Gayoso A., Lopez R., Xing G., Boyeau P., Valiollah Pour Amiri V., Hong J., Wu K., Jayasuriya M., Mehlman E., Langevin M., Liu Y., Samaran J., Misrachi G., Nazaret A., Clivio O., Xu C., Ashuach T., Lotfollahi M., Svensson V., Veiga Beltrame E., Kleshchevnikov V., Talavera-Lopez C., Pachter L., Theis F.J., Streets A., Jordan M.I., Regier J., Yosef N.: A python library for probabilistic analysis of single-cell omics data. Nat. Biotechnol. 40(2), 163–166 (2022) 10.1038/s41587-021-01206-w 35132262

[75] Gao T., Soldatov R., Sarkar H., Kurkiewicz A., Biederstedt E., Loh P.-R., Kharchenko P.V.: Haplotype-aware analysis of somatic copy number variations from single-cell transcriptomes. Nat. Biotechnol. 41(3), 417–426 (2023) 10.1038/s41587-022-01468-y 36163550 PMC10289836

[76] Lange M., Bergen V., Klein M., Setty M., Reuter B., Bakhti M., Lickert H., Ansari M., Schniering J., Schiller H.B., Pe’er D., Theis F.J.: Cellrank for directed single-cell fate mapping. Nat. Methods 19(2), 159–170 (2022) 10.1038/s41592-021-01346-6 35027767 PMC8828480

[77] Setty M., Kiseliovas V., Levine J., Gayoso A., Mazutis L., Pe’er D.: Characterization of cell fate probabilities in single-cell data with palantir. Nat. Biotechnol. 37(4), 451–460 (2019) 10.1038/s41587-019-0068-4 30899105 PMC7549125

[78] Haghverdi L., Büttner M., Wolf F.A., Buettner F., Theis F.J.: Diffusion pseudotime robustly reconstructs lineage branching. Nat. Methods 13(10), 845–848 (2016) 10.1038/nmeth.3971 27571553

[79] Bergen V., Lange M., Peidli S., Wolf F.A., Theis F.J.: Generalizing rna velocity to transient cell states through dynamical modeling. Nat. Biotechnol. 38(12), 1408–1414 (2020) 10.1038/s41587-020-0591-3 32747759

[80] Gayoso A., Weiler P., Lotfollahi M., Klein D., Hong J., Streets A., Theis F.J., Yosef N.: Deep generative modeling of transcriptional dynamics for rna velocity analysis in single cells. Nat. Methods 21(1), 50–59 (2024) 10.1038/s41592-023-01994-w 37735568 PMC10776389

[81] Subramanian A., Tamayo P., Mootha V.K., Mukherjee S., Ebert B.L., Gillette M.A., Paulovich A., Pomeroy S.L., Golub T.R., Lander E.S., Mesirov J.P.: Gene set enrichment analysis: a knowledge-based approach for interpreting genome-wide expression profiles. Proc. Natl. Acad. Sci. U.S.A. 102(43), 15545–15550 (2005) 10.1073/pnas.0506580102 16199517 PMC1239896

[82] Barbie D.A., Tamayo P., Boehm J.S., Kim S.Y., Moody S.E., Dunn I.F., Schinzel A.C., Sandy P., Meylan E., Scholl C., Fröhling S., Chan E.M., Sos M.L., Michel K., Mermel C., Silver S.J., Weir B.A., Reiling J.H., Sheng Q., Gupta P.B., Wadlow R.C., Le H., Hoersch S., Wittner B.S., Ramaswamy S., Livingston D.M., Sabatini D.M., Meyerson M., Thomas R.K., Lander E.S., Mesirov J.P., Root D.E., Gilliland D.G., Jacks T., Hahn W.C.: Systematic rna interference reveals that oncogenic kras-driven cancers require tbk1. Nature 462(7269), 108–112 (2009) 10.1038/nature08460 19847166 PMC2783335

[83] Merico D., Isserlin R., Stueker O., Emili A., Bader G.D.: Enrichment map: a network-based method for gene-set enrichment visualization and interpretation. PLoS One 5(11), 13984 (2010) 10.1371/journal.pone.0013984 PMC2981572 21085593

