# Supplementary material for: ChatSpatial: Schema-Enforced Agentic Orchestration for Reproducible and Cross-Platform Spatial Transcriptomics
Source: bioRxiv. 2026 Mar 9:2026.02.26.708361. Preprint. [Version 2] doi: 10.64898/2026.02.26.708361 (PMC13014149; doi:10.64898/2026.02.26.708361)
Supplement: Supplement 1 [file NIHPP2026.02.26.708361v2-supplement-1.pdf]

bioRxiv preprint doi: https://doi.org/10.64898/2026.02.26.708361; this version posted March 9, 2026. The copyright holder for this preprint (which was not certified by peer review) is the author/funder, who has granted bioRxiv a license to display the preprint in perpetuity. It is made available under aCC-BY 4.0 International license.

Supplementary Methods

S1 Detailed MCP Architecture

Protocol Architecture: The MCP architecture follows a client-host-server design where each host can run multiple client instances, enabling modular integration of AI capabilities while maintaining clear security boundaries. This architecture is built on three core components: (1) Host: the application process (e.g., Claude Desktop, IDE) that acts as the container and coordinator, creating and managing multiple client instances, enforcing security policies and user authorization, coordinating AI/LLM integration, and aggregating context across clients; (2) Client: protocol-handling instances that maintain isolated 1:1 connections with servers, each establishing one stateful session per server, handling protocol negotiation and capability exchange, routing messages bidirectionally, and maintaining security boundaries between servers; and (3) Server: independent processes that expose specialized capabilities (tools, resources, prompts) via MCP primitives, operating with focused responsibilities while respecting security constraints, and can be either local processes or remote services. In the ChatSpatial implementation, Claude Desktop acts as the Host, the MCP client library manages individual server connections, and the ChatSpatial server exposes bioinformatics analysis capabilities. This separation ensures that servers remain simple to implement and highly composable, while the host handles complex orchestration—a key design principle where servers cannot access the full conversation history or "see into" other servers, with all cross-server coordination controlled by the host process.

Interaction Primitives: MCP defines three core interaction primitives that distribute control between participants: (1) Tools: model-driven executable functions that the LLM can invoke autonomously to perform actions (e.g., data analysis, visualization); (2) Resources: application-driven structured data sources that provide context to LLM interactions (e.g., dataset metadata, analysis results); and (3) Prompts: user-driven predefined templates for specific workflows (e.g., "analyze spatial expression," "find cell types"). The 2025 MCP specification extends these with additional capabilities including Sampling (enabling servers to request LLM completions), Roots (filesystem context provision), and Elicitation (structured data collection from users). ChatSpatial's implementation leverages Resources through two mechanisms: (a) File URI-based Image Delivery: visualization tools return file paths as text for all images, following MCP 2025 best practice of preferring URIs over embedded content. This approach avoids the $\sim$3.3$\times$ token overhead inherent to ImageContent encoding (where a 16KB image becomes 53KB in the MCP response), optimizing context window usage while supporting multiple export formats (PNG, PDF, SVG, EPS, TIFF) for publication-quality figures; and (b) Standalone Resources infrastructure: `SpatialResourceManager` and `SpatialPromptManager` are implemented internally for dataset metadata exposure and workflow templates, currently dormant but available for activation through decorator registration (`@mcp.list_resources`, `@mcp.read_resource`). The platform exposes 20 analytical tools via the Tools primitive through MCP capability negotiation.

Communication Layer: All communication follows the JSON-RPC 2.0 specification with strict lifecycle management. The protocol defines three phases: (1) Initialization:

bioRxiv preprint doi: https://doi.org/10.64898/2026.02.26.708361; this version posted March 9, 2026. The copyright holder for this preprint (which was not certified by peer review) is the author/funder, who has granted bioRxiv a license to display the preprint in perpetuity. It is made available under aCC-BY 4.0 International license.

the client sends an `initialize` request declaring its protocol version and capabilities (e.g., sampling support, roots provision), the server responds with its own capabilities (e.g., tools, resources, prompts), and upon successful negotiation the client sends an `initialized` notification to begin operation; (2) Operation: bidirectional message exchange where both parties respect negotiated capabilities, using only features explicitly declared during initialization; and (3) Shutdown: graceful termination via transport-specific mechanisms. ChatSpatial implements this lifecycle through FastMCP, exposing 20 analytical tools in its capabilities declaration. The transport mechanism uses stdio (standard input/output) for local deployments and Claude Desktop integration, with JSON-RPC messages delimited by newlines. This capability-based negotiation ensures explicit feature agreement between client and server, supporting protocol extensibility while maintaining backwards compatibility.

Bioinformatics Extensions: Our MCP implementation includes bioinformatics-specific conventions for spatial transcriptomics data. The system maintains a standardized `AnnData` object format with spatial coordinates stored in `adata.obsm['spatial']` and analysis results stored in `adata.obs` or `adata.uns`, ensuring consistent data handling across all analytical tools and preserving provenance throughout multi-step workflows.

LLM Models and MCP Server Instructions: All development and testing were performed using Anthropic's Claude Sonnet 4.5 model [18]. ChatSpatial leverages MCP Server Instructions, which are injected into the LLM's system prompt at session initialization to provide workflow guidance. These instructions complement the comprehensive parameter documentation embedded in tool schemas (Section 4.3) by focusing on tool relationships, operational constraints, and platform-specific recommendations. The server instructions are structured into five key components:

(1) *Core Workflow Pattern:* Standard analysis sequence of `load_data()` → `preprocess_data()` → analysis tools → `visualize_data()`, establishing the expected operational flow.

(2) *Critical Operational Constraints:* Essential knowledge not obvious from individual tool schemas, such as preservation of raw data in `adata.raw` during preprocessing and the automatic utilization of `adata.raw` for cell communication analysis to access comprehensive gene coverage, along with species-specific parameter requirements.

(3) *Platform-Specific Guidance:* Technology-aware recommendations such as deconvolution requirements for spot-based platforms (Visium, Slide-seq) versus single-cell platforms (MERFISH, Xenium, CosMx), and method selection based on data characteristics (SpaGCN for Visium with histology images, STAGATE/GraphST for high-resolution data without images).

(4) *Tool Relationships:* Inter-tool dependencies such as spatial domain identification enabling spatial statistics, cell type annotation being required for cell communication analysis, and integration being recommended before cross-sample comparative analyses.

(5) *Parameter Guidance Philosophy:* Explicit direction to refer to tool schemas for detailed parameter documentation, avoiding duplication while maintaining the documentation-driven approach described in Section 4.3.

bioRxiv preprint doi: https://doi.org/10.64898/2026.02.26.708361; this version posted March 9, 2026. The copyright holder for this preprint (which was not certified by peer review) is the author/funder, who has granted bioRxiv a license to display the preprint in perpetuity. It is made available under aCC-BY 4.0 International license.

This two-layer guidance system—MCP Server Instructions for high-level workflows and tool schemas for parameter-level details—enables the LLM to orchestrate complex multi-step analyses while maintaining scientific rigor. The instructions emphasize that analysis continuity requires preserving `data_id` across operations, enabling the LLM to maintain session state for iterative exploration. The complete server instructions are provided verbatim below and in the source code repository.

S1.1 Complete MCP Server Instructions (Verbatim)

The following are the exact instructions injected into Claude's system prompt at session initialization, guiding how the LLM interacts with ChatSpatial's analytical tools:

```
ChatSpatial provides spatial transcriptomics analysis through
60+ integrated methods across 15 analytical categories.

CORE WORKFLOW PATTERN:
1. Always start with load_data() to import
   spatial transcriptomics data
2. Run preprocess_data() before most analytical tools (required for
   clustering, spatial analysis, etc.)
3. Use visualize_data() to inspect results after each analysis step

CRITICAL OPERATIONAL CONSTRAINTS:
- Preprocessing creates filtered gene sets for efficiency
  while preserving raw data in adata.raw
- Cell communication analysis automatically uses
  adata.raw when available for comprehensive gene coverage
- Species-specific parameters are critical: set species="mouse" or
  "human" and use appropriate resources (e.g.,
  liana_resource="mouseconsensus" for mouse)
- Reference data for annotation methods (tangram, scanvi) must be
  PREPROCESSED before use

PLATFORM-SPECIFIC GUIDANCE:
- Spot-based platforms (Visium, Slide-seq): Deconvolution is
  recommended to infer cell type compositions
- Single-cell platforms (MERFISH, Xenium, CosMx): Skip deconvolution -
  native single-cell resolution provided
- Visium with histology images: Use SpaGCN for spatial domain
  identification
- High-resolution data without images: Use STAGATE or GraphST

TOOL RELATIONSHIPS:
- Spatial domain identification -> Enables spatial statistics
  (neighborhood enrichment, co-occurrence)
```

bioRxiv preprint doi: https://doi.org/10.64898/2026.02.26.708361; this version posted March 9, 2026. The copyright holder for this preprint (which was not certified by peer review) is the author/funder, who has granted bioRxiv a license to display the preprint in perpetuity. It is made available under aCC-BY 4.0 International license.

```
- Cell type annotation -> Required for cell communication analysis
- Deconvolution results -> Can be used for downstream
  spatial statistics
- Integration -> Recommended before cross-sample
  comparative analyses

PARAMETER GUIDANCE:
All tools include comprehensive parameter documentation in their
schemas. Refer to tool descriptions for default values and
platform-specific optimizations.

For multi-step analyses, preserve data_id across operations to
maintain analysis continuity.
```

These instructions work in conjunction with the detailed parameter documentation embedded in each tool's schema (Section S3), creating a two-tier knowledge system where high-level workflow guidance is provided through server instructions and granular parameter details are accessible through MCP's schema introspection mechanism.

S2 Complete SpaGCN Integration Example

To illustrate our MCP-based integration architecture, we detail the implementation of SpaGCN (v1.2.5) spatial domain identification:

MCP Tool Layer. The entry point is exposed as an MCP tool through the `@mcp.tool()` decorator in `server.py`, registered as `identify_spatial_domains(data_id, params, context)`. This tool is accessible to LLMs through the Model Context Protocol, enabling natural language interactions like "identify spatial domains using SpaGCN with 7 clusters."

Method Dispatch Layer. The main function `identify_spatial_domains()` in `tools/spatial_domains.py` serves as a dispatcher. It validates input data, auto-detects the spatial coordinate key in `adata.obsm` (supporting common conventions like `'spatial'`, `'X_spatial'`), performs quality checks (handling NaN/inf values, validating data types), and routes to method-specific implementations based on `params.method`. For SpaGCN, it calls `_identify_domains_spagcn()`.

Algorithm Wrapper Layer. The `_identify_domains_spagcn()` function prepares data for SpaGCN's requirements: (1) extracts spatial coordinates via auto-detection, (2) loads histology images with scale factors if available (10x Visium format), (3) applies SpaGCN-specific gene filtering via `spg.prefilter_genes()` and `spg.prefilter_specialgenes()`, and (4) calls `SpaGCN.ez_mode.detect_spatial_domains_ez_mode()` with parameters `n_clusters`, `s` (histology weight), `b` (spot area for color extraction), `p` (neighborhood expression weight), and `r_seed` (random seed). The function includes timeout protection (default 600s) using asyncio thread pooling to prevent hanging on large datasets.

bioRxiv preprint doi: https://doi.org/10.64898/2026.02.26.708361; this version posted March 9, 2026. The copyright holder for this preprint (which was not certified by peer review) is the author/funder, who has granted bioRxiv a license to display the preprint in perpetuity. It is made available under aCC-BY 4.0 International license.

Result Standardization. Domain labels are stored in `adata.obs['spatial_domains_spagcn']` as categorical variables. An optional refinement step applies spatial smoothing using k-nearest neighbors (following SpaGCN's post-processing approach) to reduce noise while preserving domain boundaries. The result is returned as a `SpatialDomainResult` object containing domain counts, statistics, and visualization-ready metadata.

S3 Examples of LLM-Visible Parameter Documentation

ChatSpatial achieves intelligent parameter selection through comprehensive documentation visible to the LLM via MCP's schema system, rather than hard-coded rule engines. This design philosophy enables flexible, context-aware parameter optimization while maintaining transparency and user control.

Documentation-Driven Parameter Selection: All parameters are defined using Pydantic models with extensive `Field()` descriptions that the LLM can access through MCP tool schemas. For example, the `normalization` parameter includes detailed guidance on available methods (log, pearson_residuals, none), technology-specific recommendations (Visium vs. MERFISH vs. Xenium), requirements and constraints, error handling behavior, and scientific rationale. Similarly, the `normalize_target_sum` parameter provides decision guides: "None (default) uses median counts for adaptive behavior; 1e4 (10,000) is standard for 10x Visium; 1e6 (1,000,000) is standard for MERFISH/CosMx/Xenium." This documentation-first approach enables the LLM to make informed parameter choices based on the user's data characteristics and research goals without requiring pre-programmed rules.

Comprehensive Tool-Level Guidance: Every MCP tool exposes rich docstrings through the `@mcp.tool()` decorator. For instance, the `preprocess_data()` tool provides detailed notes on normalization methods, adaptive configuration options (e.g., "n_neighbors: None = adaptive based on dataset size"), and important data handling behaviors (e.g., preservation of raw data in `adata.raw` for downstream analyses). These docstrings serve as in-context guidance, allowing the LLM to understand method trade-offs and make appropriate parameter recommendations during conversations.

Conversational Error-Driven Refinement: When parameter-related errors occur, the structured error messages returned by ChatSpatial's decorator-based error handling system enable the LLM to diagnose issues and adjust parameters conversationally. For example, if a normalization method fails due to data type incompatibility (e.g., `pearson_residuals` requires raw integer counts), the error message explicitly states requirements and suggests alternatives. The LLM observes these errors and can propose corrected parameters based on the comprehensive documentation it has access to, creating an iterative refinement loop that does not require pre-defined rules for every possible failure scenario.

Default Values from Best Practices: Parameter defaults are derived from systematic review of method-specific tutorials, published best practices, and benchmark studies in the spatial transcriptomics literature. For example, clustering resolution defaults to 1.0 (matching scanpy convention), `n_hvgs` defaults to 2000 (balancing computational efficiency with information retention), and SpaGCN spatial smoothing parameter `s`

bioRxiv preprint doi: https://doi.org/10.64898/2026.02.26.708361; this version posted March 9, 2026. The copyright holder for this preprint (which was not certified by peer review) is the author/funder, who has granted bioRxiv a license to display the preprint in perpetuity. It is made available under aCC-BY 4.0 International license.

defaults to 1.0 (recommended by the original authors). These empirically validated defaults serve as starting points that the LLM can adjust based on user feedback or error messages, rather than being dynamically computed from dataset characteristics.

S4 Error Handling Code Implementation

ChatSpatial implements a structured error handling system designed for conversational interaction. The core principle is that errors should be informative and actionable, enabling the LLM to guide users toward solutions through natural dialogue rather than requiring manual debugging.

S4.1 Semantic Exception Hierarchy

Rather than relying on generic Python exceptions, ChatSpatial defines a semantic exception hierarchy (`utils/exceptions.py`) that immediately communicates error categories:

```
ChatSpatialError (base)
+-- DataError (data access/format issues)
|   +-- DataNotFoundError (required data missing)
|   +-- DataCompatibilityError (format/species mismatch)
+-- ParameterError (invalid user input)
+-- ProcessingError (algorithm/computation failures)
+-- DependencyError (missing packages/R environment)
```

This hierarchy enables precise error handling: `DataNotFoundError` for missing datasets, `ParameterError` for invalid parameter values, and `DependencyError` for missing R packages. Each exception type carries semantic meaning that the LLM can interpret to provide targeted guidance. For example, a `DataCompatibilityError` indicating gene naming mismatch prompts the LLM to suggest checking whether both datasets use the same naming convention (Ensembl vs. Symbol), while a `DependencyError` triggers installation instructions for the required package.

S4.2 MCP Error Handler Decorator

The `@mcp_tool_error_handler` decorator (`utils/mcp_utils.py`) wraps all MCP tools to ensure errors are returned as structured results rather than raised as unhandled exceptions. This allows the LLM to observe failures and respond conversationally:

```
@mcp_tool_error_handler()
async def analyze_data(...):
    # Tool implementation
```

The decorator performs runtime type inspection via `typing.get_type_hints()` to determine the appropriate error format for each tool's return type: BaseModel results re-raise for FastMCP schema handling, string-returning tools (e.g., visualization file

bioRxiv preprint doi: https://doi.org/10.64898/2026.02.26.708361; this version posted March 9, 2026. The copyright holder for this preprint (which was not certified by peer review) is the author/funder, who has granted bioRxiv a license to display the preprint in perpetuity. It is made available under aCC-BY 4.0 International license.

paths) return error messages as strings, and other types return structured error dictionaries with optional tracebacks. This type-aware approach maintains MCP protocol consistency while providing clear, actionable feedback to the LLM.

Complete implementation details, including input validation patterns and result caching strategies, are available in the source code repository.

bioRxiv preprint doi: https://doi.org/10.64898/2026.02.26.708361; this version posted March 9, 2026. The copyright holder for this preprint (which was not certified by peer review) is the author/funder, who has granted bioRxiv a license to display the preprint in perpetuity. It is made available under aCC-BY 4.0 International license.

Table S1: Integrated method library in ChatSpatial. For each of the 60+ integrated methods, we list its primary analytical category, the specific version of the software package used in our implementation, and the primary citation for the original work.

Analytical Category	Method Name	Version	Primary Citation
Data Loading & Preprocessing	Scanpy	1.9.3	[43]
	SpatialData	0.2.0	[44]
	SCTransform	0.4.0	[45]
Multi-sample Integration	Harmony	0.0.9+	[29]
	scVI	1.0.0+	[30]
	BBKNN	1.5.0+	[46]
	Scanorama	1.7.0+ (optional)	[47]
Spatial Registration	PASTE	1.3.0	[48]
	STalign	2.0.0	[49]
Visualization	Squidpy	1.3.0	[36]
	Matplotlib/ Seaborn	3.7.1/0.12.2	[50]
Cell Type Annotation	Tangram	1.0.3	[51]
	scANVI	1.0.3	[52]
	CellAssign	0.1.0	[53]
	mLLMCellType	1.0.0	[54]
	scType	via R	[55]
	SingleR	via R	[56]
Differential Expression	Wilcoxon	via scanpy	[43]
	t-test	via scanpy	[43]
	logreg	via scanpy	[43]
Spatial Domain Identification	SpaGCN	1.2.5	[57]
	STAGATE	1.1.0	[58]
	GraphST	1.1.0+	[59]
	Leiden	via scanpy	[60]
	Louvain	via scanpy	[61]

Continued on next page

bioRxiv preprint doi: https://doi.org/10.64898/2026.02.26.708361; this version posted March 9, 2026. The copyright holder for this preprint (which was not certified by peer review) is the author/funder, who has granted bioRxiv a license to display the preprint in perpetuity. It is made available under aCC-BY 4.0 International license.

Table S1 – continued from previous page

Analytical Category	Method Name	Version	Primary Citation
Spatially Variable Genes	SPARK-X	1.3.0	[62]
	SpatialDE	1.1.3	[63]
Cell-Cell Communication	LIANA+	1.0.0	[64]
	CellPhoneDB	via LIANA	[65]
	CellChat	native R	[66]
	FastCCC	0.1.0	[67]
Deconvolution	FlashDeconv	0.1.0	[68]
	RCTD	2.2.0	[34]
	Cell2location	0.1.3	[69]
	SPOTlight	0.1.7	[70]
	Stereoscope	0.3.0	[71]
	DestVI	0.1.0	[72]
	Tangram (mapping)	1.0.3	[51]
	CARD	2.0.0	[73]
CNV Analysis	inferCNVpy	0.6.1	[74]
	Numbat	1.3.2	[75]
Trajectory Inference	CellRank	2.0.0	[76]
	Palantir	1.3.0	[77]
	DPT	via scanpy	[78]
RNA Velocity	scVelo	0.2.5	[79]
	VeloVI	1.0.0	[80]
Enrichment Analysis	GSEApy	1.0.6	[81]
	Pathway GSEA	via GSEApy	[81]
	Pathway ORA	via GSEApy	[81]
	ssGSEA	via GSEApy	[82]
	EnrichMap	0.1.10	[83]
Spatial Statistics	Moran's I	via squidpy	[36]
	Local Moran's I (LISA)	via squidpy	[36]

Continued on next page

bioRxiv preprint doi: https://doi.org/10.64898/2026.02.26.708361; this version posted March 9, 2026. The copyright holder for this preprint (which was not certified by peer review) is the author/funder, who has granted bioRxiv a license to display the preprint in perpetuity. It is made available under aCC-BY 4.0 International license.

Table S1 – continued from previous page

Analytical Category	Method Name	Version	Primary Citation
	Geary's C	via squidpy	[36]
	Getis-Ord Gi*	via esda/ PySAL	[36]
	Neighborhood Enrichment	via squidpy	[36]
	Co-occurrence	via squidpy	[36]
	Ripley's K/L	via squidpy	[36]
	Centrality Scores	via squidpy	[36]
	Bivariate Moran's I	4.6.0	[36]
	Join Count Statistics	via esda/ PySAL	[36]
	Local Join Count	via esda/ PySAL	[36]
	Network Properties	via squidpy	[36]
	Spatial Centrality	2.6.0	[36]

Table S2: Comprehensive test scenarios used to validate the robustness and functionality of the ChatSpatial platform. Each scenario was designed to test a specific aspect of the platform, from handling diverse data types to executing complex multi-method workflows.

ID	Scenario	Input Data	Command /Goal	Expected Outcome	Res.	Notes
Category 1: Data Handling & Preprocessing						
1	Standard Visium Input	Visium, 4,992 spots, h5ad	"Load and preprocess the Visium data."	Data loaded, QC metrics calculated.	Pass	Standard workflow.
2	High-res Xenium Input	Xenium, 150k cells	"Load Xenium data and create AnnData."	Data loaded, valid AnnData created.	Pass	Non-h5ad input.
3	Low-quality Data	Visium, <500 genes/spot	"Filter low-quality spots."	Low-quality spots removed.	Pass	QC validation.
4	Multi-sample Integration	3 Visium slides	"Integrate samples with Harmony."	Batch effects reduced.	Pass	Integr. test.
5	Missing Spatial Coords	No spatial coordinates	"Plot spatial MALAT1."	Error: "No spatial coordinates."	Pass	Error handling.
Category 2: Core Spatial Analysis						

Continued on next page

bioRxiv preprint doi: https://doi.org/10.64898/2026.02.26.708361; this version posted March 9, 2026. The copyright holder for this preprint (which was not certified by peer review) is the author/funder, who has granted bioRxiv a license to display the preprint in perpetuity. It is made available under aCC-BY 4.0 International license.

Table S2 – continued from previous page

ID	Scenario	Input Data	Command /Goal	Expected Outcome	Res.	Notes
6	Basic Clustering	DLPFC Visium (spatialLIBD)	"Identify spatial domains using SpaGCN."	Anatomical layers identified.	Pass	Core function.
7	High-density Clustering	MERFISH, 50k cells	"Use GraphST for fine domains."	Sub-clusters identified.	Pass	Dense data test.
8	Spatial Variable Genes	Mouse brain	"Find top 50 SVGs with SPARK-X."	Known markers ranked high.	Pass	SVG detection.
9	Cell Deconvolution	Visium + scRNA ref	"Deconvolve cell types using RCTD."	Cell proportions generated.	Pass	Deconv.
10	Cell Annotation	Xenium + atlas	"Annotate cells with Tangram."	Major types assigned.	Pass	Annot. test.
11	Trajectory Analysis	Dev. tissue data	"Infer trajectory using CellRank."	Bio. pathway identified.	Pass	Advanced test.
12	RNA Velocity	Visium + spliced counts	"Calculate spatial RNA velocity."	Dynamics visualized.	Pass	Complex analysis.
13	Spatial Statistics	Mouse brain Visium	"Compute Moran's I for top marker genes."	Spatial autocorrelation scores generated.	Pass	Statistics test.
14	Pathway Enrichment	Cancer Visium	"Run GSEA on cluster markers using GO database."	Enriched pathways identified.	Pass	Enrich test.
15	CNV Detection	Tumor Visium + ref	"Infer CNV using infercnvpy with immune cells as reference."	CNV profiles generated.	Pass	CNV test.
16	Spatial Registration	Serial sections	"Align 3 consecutive slices with PASTE."	Slices registered to common space.	Pass	Reg test.
Category 3: Conversational Workflows						
17	Iterative Analysis	Tumor microenv.	"Find clusters. Now, markers for cluster 3."	Context preserved.	Pass	Context test.
18	Ambiguous Command	Any dataset	"Analyze the data."	Clarification requested.	Pass	Proactive help.
19	Parameter Override	Visium data	"Run STAGATE with resolution 1.5."	User params used.	Pass	User control.
20	Cross-category Flow	Cancer + ref	"Cluster, then find tumor-immune comm."	Sequential execution.	Pass	Workflow test.
21	Error Recovery	Large dataset	"Run cell communication analysis."	Alternatives suggested.	Pass	Error handling.

Continued on next page

bioRxiv preprint doi: https://doi.org/10.64898/2026.02.26.708361; this version posted March 9, 2026. The copyright holder for this preprint (which was not certified by peer review) is the author/funder, who has granted bioRxiv a license to display the preprint in perpetuity. It is made available under aCC-BY 4.0 International license.

Table S2 – continued from previous page

ID	Scenario	Input Data	Command /Goal	Expected Outcome	Res.	Notes
Category 4: Scalability & Stress Tests						
22	Large Dataset	100k+ Visium HD	"Run full analysis pipeline."	Pipeline completed.	Pass	Scale.
23	Small Dataset	300-spot STARmap	"Cluster the data."	Runs despite noise.	Pass	Edge case.
24	Sparse Genes	>99.9% sparsity	"Find variable genes."	Warning issued.	Pass	Robust.
25	Noisy Reference	Ambiguous scRNA ref	"Annotate using noisy ref."	Low confidence scores.	Pass	Quality aware.
26	Concurrent Users	2 users, diff. data	U1: "Cluster brain." U2: "Annotate tumor."	No interference.	Pass	Isolation test.
27	Long Context	20+ commands	"Result of first clustering?"	History retrieved.	Pass	Memory test.
28	Invalid Input	Non-scientific cmd	"Write Python code to download a file from the internet."	Polite refusal; redirected to spatial analysis scope.	Pass	Scope boundary test.

Table S3: Comparison of ChatSpatial with other AI agents for spatial transcriptomics. This table summarizes architectural design choices, not empirical performance benchmarks. ChatSpatial and STAgent provide complete open-source implementations on GitHub (MIT license); SpatialAgent's source code has not been released at the time of writing.

Feature	ChatSpatial (This Work)	STAgent	SpatialAgent
**Primary Philosophy**	Agentic Orchestrator. Reliably executes user-defined strategies with scientist in full control.	Autonomous Discovery Agent. End-to-end analysis with minimal human input.	Hybrid Autonomous Agent. Fully autonomous or interactive co-pilot mode.
**Planning & Execution**	Schema-Enforced Tool-calling via MCP.	Dynamic Code Generation via RAG.	Hybrid: predefined templates + *de novo* code generation.
**Reprod.**	Near-deterministic. 100% tool selection consistency across 240 trials (3 LLMs, $T = 1.0$); 75.7% constrained parameter consistency.	Non-deterministic. LLM code generation may vary between runs.	Mode-dependent. Deterministic for templates; variable for generated plans.

Continued on next page

bioRxiv preprint doi: https://doi.org/10.64898/2026.02.26.708361; this version posted March 9, 2026. The copyright holder for this preprint (which was not certified by peer review) is the author/funder, who has granted bioRxiv a license to display the preprint in perpetuity. It is made available under aCC-BY 4.0 International license.

Table S3 – continued from previous page

Feature	ChatSpatial (This Work)	STAgent	SpatialAgent
**Tool Scope**	Cross-Ecosystem (Python & R). 60+ methods.	Python-Centric. Scanpy/Squidpy.	Python-Centric. 19 curated tools.
**Extensibility**	Open standard (MCP). New tools added by defining schema.	Internal codebase. Requires re-indexing RAG database.	Internal framework. Architecture details pending code release.
